# E. B. Wolcott, M. D.

**Published:** 1880-02

**Authors:** 


### Obituary.

### SURGEON-GENERAL E. B. WOLCOTT.

Dr. Erastus B. Wolcott, Surgeon-General of the State of Wisconsin, one of the most widely known men of the Northwest, died at his residence on Milwaukee street at 11 o'clock Jan. 5th, after an illness of one week. There are few men whose death would cause more general sorrow; few men have worked as faithfully and unselfishly, as long and laboriously, in one of the most noble of professions, as Dr. Wolcott. The announcement made this morning will carry pain to hundreds of households where the cheerful face of Dr. Wolcott has brought hope and his skill has brought relief from suffering.

Erastus B. Wolcott, M.D., was born at Benton, Yates county, New York, October 18, 1804, son of Elisha and Ann Hull Wolcott, who came from Litchfield county, Connecticut and were among the first settlers in that section of country. In 1822 Dr. Wolcott commenced the study of medicine and surgery with Dr. Joshua Lee, an eminent physician and surgeon of Central New York, and received a diploma from Yates County Medical Society in 1825. He attended the College of Physicians and Surgeons of the Western District of New York from 1830 to 1833, and took his degree in medicine and surgery at that institution. In the spring of 1835 he was examined by a board of army surgeons and received an appointment as surgeon in the United States army, Jan. 1, 1836. He resigned in 1839 and came to Milwaukee. In 1836 he married Elizabeth J. Dousman, who died in 1860, leaving a daughter and a son. Dr. Wolcott gave his children a liberal education, the former having graduated at Milwaukee Female college, and the latter at Yale college.

Dr. Wolcott was connected with some of the earliest enterprises of the State. He built the first mills at West Bend, and with others the first mill at Humboldt, near the city. He was one of the prime movers in building the first railroad in the State, from Milwaukee to the Mississippi River, and among the first in the Northwestern life insurance company. He was appointed trustee of the Wisconsin Hospital for the Insane, the first year, and re-appointed through Gov. Randall's and Gov. Lewis' administrations. He was appointed one of the regents of the State university in 1850. He was appointed surgeon of the State militia as early as 1842, by Gov. Doty, was commissioned colonel of a regiment in 1846, and in the same year major-general of the first division of Wisconsin militia. He held through the war of the rebellion the position of surgeon-general of Wisconsin, with the rank of brigadier-general, which he retained to the day of his death. In 1866 Dr. Wolcott was appointed by Gov. Fairchild commissioner to represent Wisconsin at the universal exposition at Paris in 1877. In the same year he was appointed by Congress manager of the National Home for Disabled Volunteers, near this city, to which position he was re-appointed in 1875.

Dr. Wolcott was married Oct. 12, 1869, to Laura J. Ross, M. D., who was among the first women who graduated in medicine and received hospital instruction in this country. Dr. Wolcott was a lineal descendant of Henry Wolcott, Esq., a landed gentleman of England, who came to America in 1630. He was the son and heir of John Wolcott, of Golden Manor. The manor house is still standing in England, is of great antiquity, and richly ornamented with carved work. Upon the walls may be seen the motto of the family coat of arms: *Nullius addictus jurare in verba magistri* (inclined to swear on the words of no master.) This sentiment was in harmony of the spirit of the English gentlemen of the middle ages and that of the Puritan of a later date, who spurned the dictation of ecclesiastical wisdom. This peculiarity of the family lost none of its force in the character of Dr. E. B. Wolcott, who derived his knowledge of the Author of all things from the study of His works.

Henry Wolcott, of the old English gentry, was the first magistrate in the Connecticut colony, and his descendants in a direct line for over one hundred and eighty years, were counselors of war, officers of the army during the revolution, one a signer of the Declaration of Independence, representatives and senators in congress, chief justices of the supreme court, and six governors of Connecticut, three bearing the name of Wolcott—Roger, Oliver and Oliver junior. Roger Wolcott, the first governor of Connecticut, was judge of the county court, deputy governor, chief justice of the Superior court, and governor of the State. He lived to see his son Oliver governor during fourteen years, and his grandson Oliver during four years. Of his descendants bearing the name of Wolcott, twelve were graduates of Yale college, two of Harvard university, and two at other New England colleges previous to the year 1834. Those qualities which characterized the ancestors of Dr. E. B. Wolcott, whether by hereditary descent, by example, or education, seemed to have culminated in him. His form was symmetrical, his movements graceful and almost up to the day of his death his youthful energy seemed unimpaired. His mind was vigorous and active, embracing a wide field of observation. Always eminent in his profession, he kept a steady step in the march of medical science Skilled as a surgeon, the knife never trembled in his hand. Unerring in his diagnosis, he waited with the patience of a nurse. His sensibilities were always keenly alive to every object of human suffering. As a son, husband, father and friend he discharged his duties with scrupulous fidelity. We have been told that Cervantes "smiled the chivalry of Sham away." If so, she, like Liberty, took her flight to the new world, and found worshippers in its forests. If truth, justice, honor and mercy are her characteristics they were all happily personated in the character of the late Dr. E. B. Wolcott.

A week before his death, at the solicitation of members of the profession at Horicon, Dr. Wolcott generously left his business to give testimony as an expert in a suit for malpractice. He was kept in the court-room till ten o'clock at night, subject to draughts and changes of temperature. and when he went to his hotel, found he had taken cold. The symptoms of pneumonia rapidly appeared and when he was brought home his condition was hopeless. All was done for him by his warm friend Dr. Spearman and others, that was possible, but to no purpose. He sank steadily and Monday morning, Jan. 5th, all hope was surrendered. At eleven o'clock p. m. he breathed his last, surrounded by loving friends.

The flag on the capitol was placed at half-mast in respect to the memory of Surgeon-General Wolcott, and universal regret expressed at his sudden death. The following general order was issued:

"STATE OF WISCONSIN, EXECUTIVE OFFICE, MADISON, JAN. 6, 1880.—*General Order No.* 2.—The sad duty is devolved upon the Governor of announcing to the people of Wisconsin, and especially to the officers and men of the Wisconsin National Guard, the sudden and unexpected death of Brig. Gen. Erastus B. Wolcott, M.D., which occurred at his residence in the city of Milwaukee, at 11 o'clock yesterday evening. Gen. Wolcott was born in Benton, Yates county, New York, in October, 1804. He received his diploma as a doctor of medicine in 1825. He was appointed surgeon in the United States army in 1836, and resigned in 1839, and the same year settled in Milwaukee, where he has ever since resided. In 1842 he was appointed surgeon of the territorial militia, by Gov. Doty. In 1846, colonel of a regiment, and in the same year major-general of the first division of Wisconsin militia. At the breaking out of the war of the rebellion, he was appointed to the laborious and responsible office of surgeon-general of the State, and with the exception of a brief interval of about three months, continued in the discharge of the duties of that office, with universal approbation, during the whole period of the war, and until January 5, 1874. On the 11th day of January, 1876, he was again appointed surgeon-general, and continued as such by successive appointments until relieved by death, his last commission having been issued yesterday, in the hope and expectation that his indisposition would be but temporary.

"Although prominent in his chosen profession in general practice as well as in his connection with the army and the Wisconsin militia, it was not alone in these relations that he was known and respected. In 1850 he was appointed a regent of the University of Wisconsin. In 1860 a trustee of the State Hospital for the Insane, holding for six years. In 1866 a commission to represent Wisconsin at the universal exposition in Paris; and the same year a manager of the National Home for Disabled Volunteer Soldiers, which office he held by reappointment at the date of his death.

"It may be truthfully said of Gen. Wolcott that in all these public positions, and in the wide and possibly more important sphere of a citizen, he was efficient, faithful and zealous. Broad and sympathetic in his views, polished in manner, wise in counsel, vigorous in action, the personification of honor and integrity, his death may well be mourned as a public calamity. In token of respect for his memory, the members of the staff are requested to wear the usual badge of mourning for a period of thirty days from the receipt of this order, and all officers of the Wisconsin National Guard will wear a similar badge during the same period, when in uniform. On the day of his funeral the flag upon the capitol will be displayed at half-mast, and the adjutant general will direct such guards of honor and formal escort to be furnished as may be agreeable to the family and immediate friends of the deceased. (Signed), WM. E. SMITH, Governor."

The members of the Milwaukee County Medical Society, and many of the prominent physicians of Milwaukee, met in the parlors of the Newhall House, and after somewhat extended remarks by several of the gentlemen in regard to the life and services of the late Dr. E. B. Wolcott, adopted the following resolutions:

"WHEREAS, By the will of the Divine Author of Creation, our honored associate, Dr. E. B. Wolcott, has been removed from among us, and the medical society of Milwaukee county has thereby been deprived of one of its valued members, we desire to give expression to our respect, esteem and affection for his memory; therefore,

"Resolved, That we desire as individuals and as a society to record our appreciation of his life and character, of his worth as a scholarly gentleman, and our admiration of his noble qualities of head and heart.

"Resolved, That more than fifty years of professional labor, mostly in the State of Wisconsin, have won for him a wide-spread confidence and many honors justly conferred, and a distinguished place among the surgeons of the country.

"Resolved, That in the life, labor and character of Dr. E. B. Wolcott we have an example of industry, manliness and usefulness, in a high degree commendable and worthy of imitation.

"Resolved, That in the death of Dr. Wolcott this society, the medical profession of this city, and society in general have sustained a great loss.

"Resolved, That this society feel that words are inadequate to express the full sense of this bereavement, but desire to record the estimation in which the deceased was held, whose memory they will cherish with the sincerest affection.

"Resolved, That we tender to the family of Dr. Wolcott our profound sympathy in their bereavement.

"Resolved, That these resolutions be recorded in the minutes of this society, and a copy be sent to the family of the deceased."

The society voted to attend the funeral in a body.

At a meeting of the Milwaukee Academy of Medicine, convened to give expression of sentiment in regard to the recent death of Dr. Wolcott, of this city, the following resolutions were unanimously adopted:

"Resolved, That in the death of Dr. E. B. Wolcott, Surgeon General of the State, the people have lost a true and generous friend, and the profession an elder brother, whose skillful hand was ever extended at the call of distress and suffering, knowing no distinctions of wealth among his patients, no limits save those of honor in his professional associations. The name of our departed friend will ever remain a revered one in Wisconsin homes and medical gatherings. The officers of this society are hereby instructed to communicate to his family this expression of our regard; and to invite the homeopathic physicians of Milwaukee to attend his funeral in a body, as a mark of respect and esteem for one whose heart rose above all distinctions of sect, party, or pathy, and whose last professional act was in defense of a homeopathic physician.

The following general order was issued from Washington, D. C.:

*Head-Quarters National Home for Disabled Volunteer Soldiers.*

WASHINGTON, D. C., January 6, 1880.

The telegraph brings the mournful news of the death of Manager Erastus B. Wolcott, of Wisconsin.

It is fitting when a good man dies that those associated with him, at least, should pause in their pursuits, in reverence to his memory. It is therefore ordered:

*First.* That on the day of his funeral, guns shall be fired half-hourly from sunrise until the hour thereof, at each Branch.

*Second.* That the flags shall be at half-mast from the receipt of this order, including the day of the funeral.

*Third.* That the several officers of the Home shall wear the usual badge of mourning for thirty days.

*Fourth.* That on the day of the funeral all ordinary avocations and business shall be suspended.

*Fifth.* General Hincks is instructed to furnish such escort, and pay such honors to the deceased as may be acceptable to his family, and will, on receipt of this order, give notice to the other Branches of the day and hour of the funeral.

*Sixth.* This order shall be read to each company in the several Branches by its sergeant on the morning of its reception.

BENJ. F. BUTLER,
*President Board of Managers.*

OFFICIAL:

EDW. W. HINCKS,

*Commandant.*

---

THE post-graduate, or practitioners' courses of medical lectures, in the colleges of this city, are promising well. That at Rush College, now in progress, is well attended by medical gentlemen, who are much pleased with the character of the instruction given. That announced for the Chicago Medical College begins on the 31st of March next, and will doubtless meet with a deserved success.

